# Multiomics analysis of the mechanisms behind flavonoid differences between purple and green tender shoots of *Camellia sinensis* var. *assamica*

**DOI:** 10.1093/g3journal/jkac297

**Published:** 2022-11-07

**Authors:** Zhi-Wei Liu, Xing-Yun Shi, Shuang-Mei Duan, Bo Nian, Li-Jiao Chen, Guang-Hui Zhang, Cai-You Lv, Yan Ma, Ming Zhao

**Affiliations:** College of Tea Science, State Key Laboratory of Conservation and Utilization of Bio-resources in Yunnan, and National & Local Joint Engineering Research Center on Germplasm Innovation & Utilization of Chinese Medicinal Materials in Southwest China, Yunnan Agricultural University, Kunming, Yunnan 650201, China; College of Tea Science, State Key Laboratory of Conservation and Utilization of Bio-resources in Yunnan, and National & Local Joint Engineering Research Center on Germplasm Innovation & Utilization of Chinese Medicinal Materials in Southwest China, Yunnan Agricultural University, Kunming, Yunnan 650201, China; Tea Technique Extension Station of the Agriculture Bureau of Dehong, Yunnan 678400, China; College of Tea Science, State Key Laboratory of Conservation and Utilization of Bio-resources in Yunnan, and National & Local Joint Engineering Research Center on Germplasm Innovation & Utilization of Chinese Medicinal Materials in Southwest China, Yunnan Agricultural University, Kunming, Yunnan 650201, China; College of Tea Science, State Key Laboratory of Conservation and Utilization of Bio-resources in Yunnan, and National & Local Joint Engineering Research Center on Germplasm Innovation & Utilization of Chinese Medicinal Materials in Southwest China, Yunnan Agricultural University, Kunming, Yunnan 650201, China; College of Tea Science, State Key Laboratory of Conservation and Utilization of Bio-resources in Yunnan, and National & Local Joint Engineering Research Center on Germplasm Innovation & Utilization of Chinese Medicinal Materials in Southwest China, Yunnan Agricultural University, Kunming, Yunnan 650201, China; College of Tea Science, State Key Laboratory of Conservation and Utilization of Bio-resources in Yunnan, and National & Local Joint Engineering Research Center on Germplasm Innovation & Utilization of Chinese Medicinal Materials in Southwest China, Yunnan Agricultural University, Kunming, Yunnan 650201, China; College of Tea Science, State Key Laboratory of Conservation and Utilization of Bio-resources in Yunnan, and National & Local Joint Engineering Research Center on Germplasm Innovation & Utilization of Chinese Medicinal Materials in Southwest China, Yunnan Agricultural University, Kunming, Yunnan 650201, China; College of Tea Science, State Key Laboratory of Conservation and Utilization of Bio-resources in Yunnan, and National & Local Joint Engineering Research Center on Germplasm Innovation & Utilization of Chinese Medicinal Materials in Southwest China, Yunnan Agricultural University, Kunming, Yunnan 650201, China

**Keywords:** flavonoids, metabolome, transcriptome, proteome, tea plant

## Abstract

Flavonoids are rich in tea plants (*Camellia sinensis*), and responsible for the flavor and healthful benefits of tea beverage. The anthocyanin levels in the purple tender shoots are higher than in the general green leaves of tea plant, which provide special materials to search metabolic mechanisms of flavonoid enrichment in plant. In this work, flavonoid differences between purple and green shoots from tea cultivars “Zijuan” (ZJ) and “Yunkang10” (YK-10) were investigated through metabolomic analysis, and mechanisms for their difference were surveyed by comparative transcriptomic and proteomic analysis. Levels of 34 flavonoids were different between ZJ and YK-10 shoots. Among them, 8 and 6 were marker metabolites in ZJ and YK-10, respectively. The differentially expressed genes (DEGs), differentially expressed proteins (DEPs), and different-level metabolites (DLMs) between ZJ and YK-10 were researched, respectively; and interactions including DEG-DLM, DEP-DLM, DEG-DEP, and DEG-DEP-DLM were analyzed; the contents of 18 characteristic flavonoids in tea leaves and expressions of 34 flavonoid metabolic genes were measured to verify the omics results. Integrated above analyses, a proposed model of flavonoids biosynthesis in tea shoots were established. The differential expression of the leucoanthocyanidin reductase (LAR), anthocyanidin synthase (ANS), anthocyanidin reductase (ANR), UDPG-flavonoid glucosyltransferase (UGT) 75L12 and 94P1 at gene level, and the ANS, ANR, and UGT78A15 at protein level, were closely associated with differences in flavonoids between ZJ and YK-10 shoot. Together, this study provides new information on the flavonoid accumulation mechanism in tea plant.

## Introduction

Flavonoids are important plant secondary metabolites that exist broadly in fruit, vegetables, spices, herbs, and beverage plants (Zhao *et al.* 2020). They produce multiple human health benefits, such as antioxidability, anti-inflammatory, anticancer, antiallergic, immunomodulatory, antihypertensive, antibacterial, and antiviral properties (Montenegro-Landívar *et al.* 2021), and their presence is associated with the bitter and astringent flavors of plant-based foods (Drewnowski 2001; McShea *et al.* 2008). Plant flavonoids are synthetized through phenylpropanoid, flavonoid, anthocyanin, flavone, and flavonol biosynthesis, and further modification of glycosylation, acylation and methylation (Chen, Shi *et al.* 2020). Many pivotal and functional enzymes are involved in the biosynthesis or modification of flavonoids, including phenylalanine ammonia-lyase (PAL), cinnamic 4-hydroxy-lase (C4H), 4-coumarate CoA ligase (4CL), chalcone synthase (CHS), chalcone isomerase (CHI), flavanone-3-hydroxylase (F3H), flavonoid synthase (FLS), flavonoid 3′-hydroxylase (F3′H), flavonoid 3′5′-hydroxylase (F3′5′H), dihydroflavonol-4-reductase (DFR), leucoanthocyanidin reductase (LAR), anthocyanidin synthase (ANS), anthocyanidin reductase (ANR), and UDPG-flavonoid glucosyltransferase (UGT) (Liu *et al.* 2021; Ma *et al.* 2021). Because flavonoids are responsible for the flavor and healthful benefits of plant foods and herbs, their biosynthesis and modifications have both scientific and commercial importance.

Tea is the most consumed beverage after water, and it is the dried product of tender tea shoots of the tea plant [*Camellia sinensis* (L.) O. Ktze]. Flavonoids, especially catechins, such as (–)-epigallocatechin-3-gallate (EGCG) and (–)-epigallocatechin (EGC), are important bioactive and flavorful substances in tea (Kim *et al.* 2014; Reygaert 2018; Davis and Running 2021). Interestingly, in many countries and regions, like China, India, Kenya, and Japan, there are some cultivars have red or purple tender shoots, such as “Zijuan” (ZJ), “Ziyan,” and “TRFK 306” (Jiang *et al.* 2013; Kerio *et al.* 2013; Joshi *et al.* 2015; Lai, Li *et al.* 2016; Maritim, Korir *et al.* 2021). They are color mutants of the tea plant with green shoot and can be inherited stably through asexual reproduction. The percentage of anthocyanins in the red or purple tender shoots is 1%–3% of dry weight, which is higher than that in green shoots (Saito *et al.* 2011; Jiang *et al.* 2013). In addition, the phenolic acids, amino acids, alkaloids and flavonoids like flavan-3-ols, proanthocyanins, flavonol, and flavone glycosides were also differentially abundant metabolites between purple and green tea leaves. The fold change values of flavonoids were obviously higher than that of other compounds (Li *et al.* 2021). Therefore, tea germplasms with purple shoots can be used for studying chemical compositions and physiological functions of anthocyanins, as well as the mechanism of flavonoid biosynthesis.

The glycosylated anthocyanins, pelargonidin 3-rhamnoside 5-glucoside, quercetin 3-rutinoside-4′-glucoside, cyanidin 3-diglucoside 5-glucoside, pelargonidin 3-sophoroside 5-glucoside, delphinidin/cyanidin-3-O-galactoside, and delphinidin/cyanidin 3-O-(6-O-p-coumaroyl) galactoside, have been identified in ZJ (Jiang *et al.* 2013; Mei *et al.* 2020; Li *et al.* 2021). Additionally, the levels of CHS, CHI, DFR, ANS, and UGT enzymes may be responsible for anthocyanin accumulation (Wang *et al.* 2017). The significantly increased genes expression levels of enzymes including UGT, ANS, anthocyanidin 3-O-glucosyltransferase, and anthocyanidin 3-O-glucoside 6″-O-acyltransferase, play important roles in increasing anthocyanin levels and their transformation (Wang, Liu *et al.* 2019; Maritim, Masand *et al.* 2021). In addition, the transcription factors MYB, bHLH, WD40, and WRKY influence anthocyanin accumulation by regulating the target genes in purple tea (Zhao *et al.* 2013; Lai, Du *et al.* 2016; Wei *et al.* 2019; Mei *et al.* 2020; Peng *et al.* 2020; Premathilake *et al.* 2020). However, the differences in the compositions and contents of flavonoids between purple and green tea leaves are not well-studied. The reasons for these differences also require further study.

Here, we explored the differences in flavonoids between the ZJ purple shoots and Yunkang-10 (YK-10) green shoots using metabolomics and high-performance liquid chromatography (HPLC) analyses. To improve the accuracy and specially explore the flavonoids biosynthesis mechanism, the targeted metabolome was chosen to analyze the flavonoids difference, both the primary and secondary spectrum data were qualitatively analyzed. The differences in expression levels of genes and enzymes involved in flavonoid biosynthesis were investigated using transcriptomics and proteomics analyses. The correlations between flavonoids and biosynthesized genes of enzymes were investigated and further verified by real-time quantitative polymerase chain reaction (RT-qPCR) and HPLC measurement. Our research increases our understanding of flavonoids and their formation mechanism in the tea plant.

## Materials and methods

### Plant materials and samples preparation

**Fig. 1.**
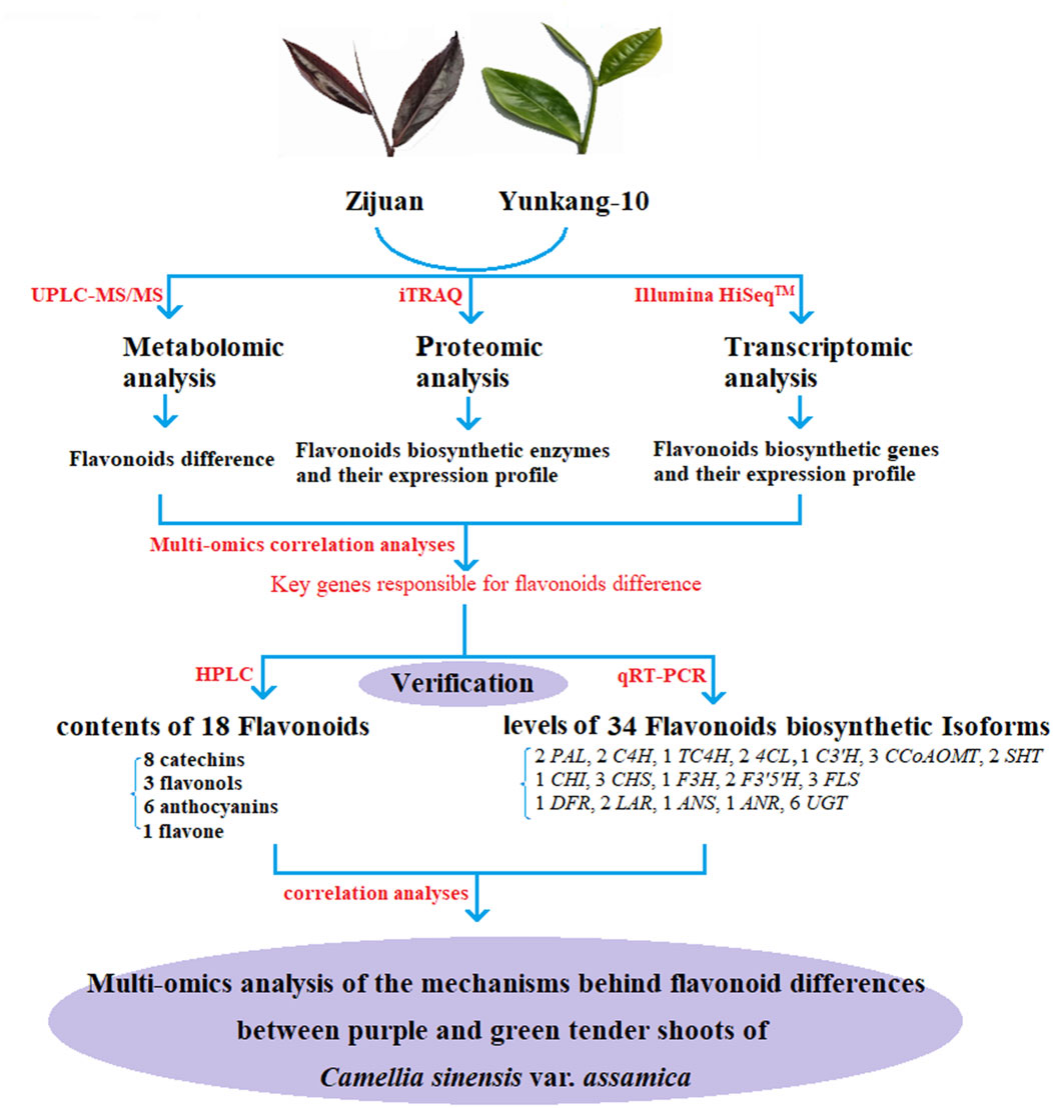
Overview of the multiomics analyses in this study.

All the plant materials were collected from the Pu’er City Institute of Tea Science (N 22°02′-24°50′, E 99°09′–102°19′), Yunnan Province, China, on September 30, 2018. Under the same picking standard (1 bud and 2 leaves), all replicate samples picked from healthy tea plants with similar growth status to ensure highly consistent. One bud and 2 leaves of purple shoots from ZJ and green shoots from YK-10 were collected for metabolomics, transcriptomics, and proteomics analyses, each with 3 biological duplications. The expression levels of 34 flavonoid biosynthetic genes were determined using RT-qPCR, and the contents of 18 tea characteristic flavonoids were measured using by HPLC (Fig. 1). The samples used for HPLC and metabolomics analyses were fixed and dried by microwave, and the samples used for transcriptomics, proteomics and RT-qPCR analyses were immediately frozen in liquid nitrogen and stored at −80°C.

### Transcriptomic analysis

Total RNA was extracted using the TRIzol method and evaluated using a NanoDrop 2000 Spectrophotometers (Thermo Fisher Scientific, Wilmington, USA) and Agilent 2100 Bioanalyzer (Agilent, California, USA). A cDNA library was constructed by amplifying full-length cDNA to generate full-length transcriptome through PacBio Isoform sequencing, as described in our previous report (Chen, Shi *et al.* 2020). Another cDNA library was constructed with amplified cDNA of greater than 300 bp to generate the 2^nd^ generation transcriptome using Illumina HiSeq2000 sequencing. High-quality isoforms or clean reads were obtained after filtration, and then were mapped to the YK-10 genome (http://www.plantkingdomgdb.com/tea_tree/) (Xia *et al.* 2017). The mapped reads were assembled using ***Cufflinks*** software and Reference Annotation Based Transcripts method (Trapnell *et al.* 2010; Roberts *et al.* 2011). Then, the assembled results of different replicate samples were merged for further analysis.

The gene expression levels were calculated using the Fragments Per Kb per Million reads (FPKM) method, and the differentially expressed genes (DEGs) were filtered using edgeR (https://bioconductor.org/packages/release/bioc/html/edgeR.html) with the criteria False Discovery Rate < 0.05 and |log_2_[Fold Change (FC)]| > 1. Based on the genes annotation description of reference genome, the DEGs were used for cluster and Gene Ontology (GO) (http://www.geneontology.org/) and Kyoto Encyclopedia of Genes and Genomes (KEGG) significant enrichment analyses (Kanehisa *et al.* 2008). The new genes (length ≥ 200 bp and exon number ≥ 2) were annotated by blasting into non-redundant proteins sequences database in NCBI (http://www.ncbi.nlm.nih.gov/) and KEGG (*E*-value < 1 × 10^−5^) database.

### Proteomic analysis

Proteins were extracted from tea shoots using the fractional precipitation method with organic solvents, and protein concentrations were measured using a Bicinchoninic Acid detection kit (P0012, Biyuntian, Shanghai, China). Extracted proteins were labeled using an iTRAQ label kit (AB SCIEX, Framingham, USA). The labeled peptides were pre-separated using high-pH reversed-phase liquid chromatography and analyzed by low-pH nano-HPLC-MS/MS (Orbitrap Fusion Tribrid, Thermo Fisher Scientific, Wilmington, USA) (Wang *et al.* 2018). Then, the MS data were analyzed using the Mascot search engine 2.3.2 (Matrix Science, London, UK). The Mascot database was established for protein identification using the tea plant genome.

According to the GO and KEGG annotations, enrichment analyses of the target proteins were fulfilled under Fisher’s exact test. The medians of relative expression levels after all “Unique Peptides” normalization were taken as the protein expression level. The differentially expressed proteins (DEPs) were filtered using the criteria |FC| >1.2 and *P <*0.05. On the basis of gene/protein expression profiles, correlations among the quantity and the GO and KEGG enrichments between the proteome and transcriptome were analyzed. The 4/9-quadrant figures were used for separating the correlated genes and proteins. To better identify the key genes/proteins, the GO and KEGG annotations of the DEPs and DEGs were further analyzed.

### Metabolomic analysis

The freeze-dried tea shoot samples were crushed into powders and extracted overnight at 4°C with 1.0 ml 70% aqueous methanol. Then, the extracts were analyzed using an LC-ESI-MS/MS system (UPLC, Shim-pack UFLC SHIMADZU CBM30A system, http://www.shimadzu.com.cn/; MS, Applied Biosystems 4500 Q TRAP, http://www.appliedbiosystems.com.cn/). The effluent was alternatively connected into an ESI-triple quadrupole-linear ion trap (Q TRAP)-MS (Chen *et al.* 2013). On the basis of the Metware database established by Metware Biotechnology Co., Ltd. (Wuhan, China), the obtained data were further processed using the software Analyst 1.6.1 (AB SCIEX). The different-level metabolites (DLMs) were filtered using the criteria VIP >1, *P <*0.05 and |log_2_(FC)| ≥1, and then, they were used for the KEGG annotation and enrichment analysis by MBRole (http://csbg.cnb.csic.cs/mbrole), cluster analysis by Hierarchy Cluster and integrated analysis by iPath2.0 (http://pathways.embl.de).

According to metabolites abundance and gene/protein expression levels, the correlations between metabolome and transcriptome/proteome were analyzed. Three models were established to analyze the correlations, the KEGG Pathways, Bidirectional orthogonal projections to latent structures and Pearson’s correlation coefficients. Furthermore, the correlations among the metabolome, proteome, and transcriptome were analyzed on the basis of the DLMs, DEPs, and DEGs involved in the flavonoid synthetic pathways.

### HPLC determination of the 18 flavonoids

The presence of 18 flavonoids, including 8 catechins [catechin (C), epicatechin (EC), catechin gallate (CG), gallocatechin (GC), EGC, epicatechin-3-gallate (ECG), gallocatechin gallate (GCG), and EGCG], 6 anthocyanins [delphinidin (Dp), cyanine (Cy), pelargonidin (Pg), peonidin (Pn), petunidin (Pt), and malvidin (Mv)], 3 flavonols [myricetin (My), quercetin (Qu), kaempferol (Kf)], and luteolin (Lu), in ZJ and YK-10 shoots were determined by HPLC (Agilent 1200, USA) with a TSKgel ODS-80TM column (4.6 mm Φ × 250 mm, 5 µm, TOSOH, Japan). In total, 8 ml of chromatographic methanol was added to 0.20 g tea-leaf powder and refluxed for 1.5 h. Then, the 10 mL diluted extract was filtered with a 0.45 µm nylon membrane. Each sample was extracted twice, and each extraction was detected 3 times.

The set conditions of the HPLC were as follow: mobile phase A was 5% acetonitrile and 0.261% phosphoric acid, phase B was 80% methanol; flow rate was 0.8 ml/min; column temperature was 35°C; injection volume was 2 μl; and the detection wavelengths were 280 nm for catechins, 530 nm for anthocyanins, and 360 nm for flavonols. The elution gradient program was 0–16 min, and B increased from 10% to 45% linearly; 16–22 min, B from 45% to 65%; 25–25.9 min, B from 65% to 100%; 25.9–29.0 min, B maintained 100%; 30 min, B decreased from 100% to 10% linearly and was then maintained at 10% for 6 min.

### RT-qPCR analysis of genes expression

The total RNA was extracted using a total RNA Extraction Kit for polysaccharide polyphenolic plants (TIANGEN, Beijing, China) and reverse transcribed to cDNA using a PrimeScript RT reagent Kit with gDNA Eraser (TaKaRa, Dalian, China). Using the TB Green Premix Ex Taq II Kit with Tli RNaseH Plus (TaKaRa), the 20-μl reaction system for RT-qPCR was established as follows: 10 μl TB Green Premix Ex Taq II (2×), 0.8 μl forward/reverse primers (10 μM), 0.4 μl ROX Reference Dye II (50×), 2 μl cDNA and 6 μl ddH_2_O.

Using the software Primer 3 Plus, the primers for RT-qPCR were designed (Supplementary Table 1). The reaction program for the RT-qPCR was as follows: 95°C pre-denaturation for 30 s, 40 cycles of 95°C denaturation for 5 s and 60°C extension for 30 s; followed by 60–95°C for a melting curve analysis. The relative expression levels of genes were calculated using the 2^–ΔΔCT^ method (Livak and Schmittgen 2001).

### Statistical analyses

The means and standard deviation represent the average values of 3 determination results, and *P <*0.05 was considered as significant difference after *t*-tests among independent samples. The SPSS 22 software (Cronk 2016) (IBM SPSS Statistics, Chicago, USA) and TBtool (Chen, Chen *et al.* 2020) were used for statistical analyses, and SIMCA14.1 software was used for PCA and OPLS-DA analysis. The heatmap, network diagram and scatter plot were constructed using Heml, Cytoscape and Excel software, respectively.

## Results

### Difference in flavonoids between shoots of ZJ and YK-10

**Fig. 2.**
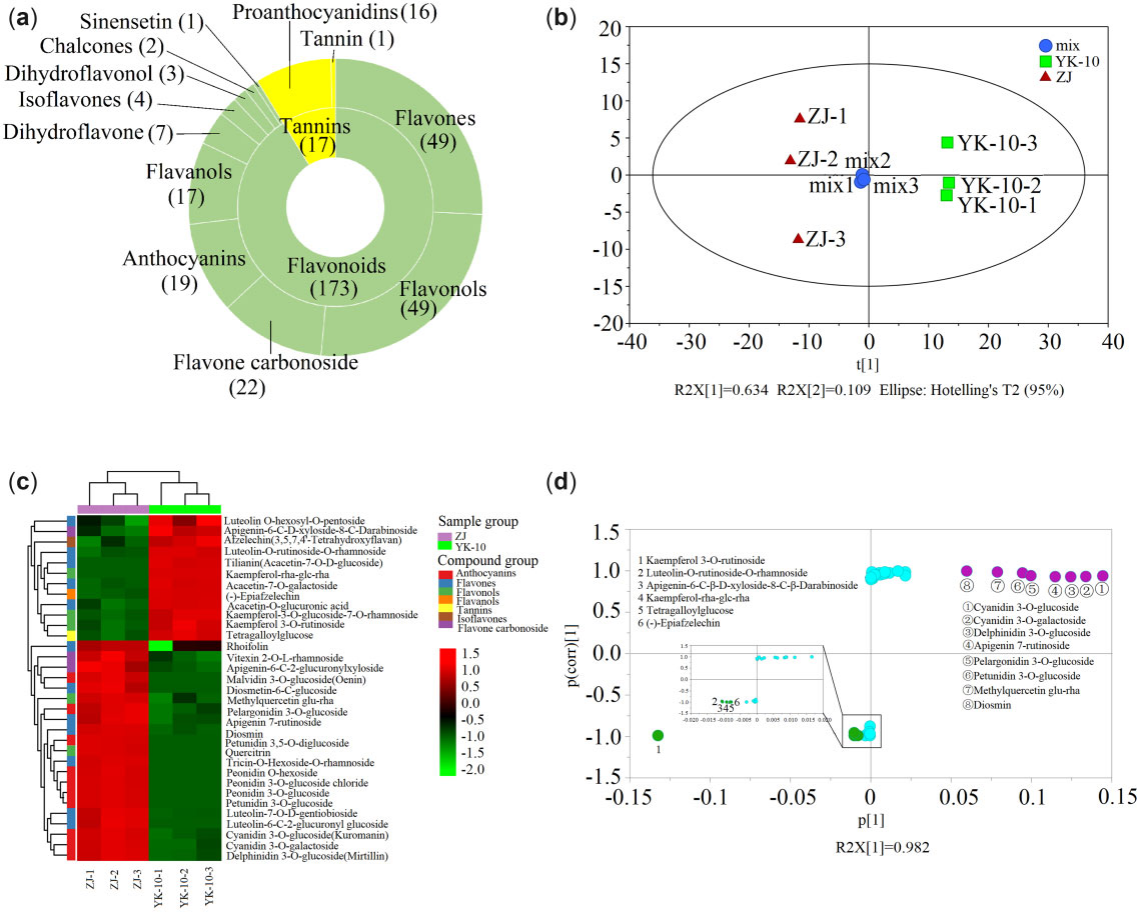
Results of metabolomics analysis. Classification of total identified flavonoids (a), PCA scores (b), heatmap (c) and S-plot (d) of 34 DLMs between ZJ and YK-10 shoots. *Note*: a: the inside circle of concentric circles represent Flavonoids and Tannins, the outside circle represent the classified compounds of Flavonoids and Tannins.

Through the UPLC-MS/MS metabolomic analysis, 187 and 178 flavonoids were identified in ZJ and YK-10 shoots, respectively. A total of 190 flavonoids and related compounds were identified, including 71 flavones and their carbon glycoside derivatives, 49 flavonols, 19 anthocyanins, 17 flavanols (catechins), 10 procyanidins, and 4 isoflavones. In the PCA, ZJ and YK-10 were grouped into different clusters, indicating that the compositions and contents of the flavonoids in ZJ and YK-10 differed (Fig. 2, a and b). In total, 20 and 14 metabolite levels were significantly higher (VIP > 1, *P <* 0.05 and FC ≥ 2) and lower (VIP > 1, *P <* 0.05 and FC < 0.5), respectively, in ZJ than in YK-10. Those flavonoids were further classified into anthocyanins, flavones, flavone oxyglycosides, flavonols, flavanols, isoflavones, and tannins. In ZJ shoots, the DLMs that were anthocyanins showed significantly higher contents, but the isoflavone (afzelechin), flavanol ((–)-epiafzelechin), and tannin (tetragalloylglucose) contents were lower than those in YK-10 (Fig. 2c; Table 1).

**Table 1.** The list of DLMs between ZJ and YK-10 shoots.

Compounds	Class II	VIP	FC	LogFC
Petunidin 3-O-glucoside	Anthocyanins	3.09	a	—
Peonidin 3-O-glucoside chloride	Anthocyanins	2.84	a	—
Peonidin 3-O-glucoside	Anthocyanins	2.84	a	—
Peonidin O-hexoside	Anthocyanins	2.82	a	—
Malvidin 3-O-glucoside (Oenin)	Anthocyanins	2.60	a	—
Petunidin 3,5-O-diglucoside	Anthocyanins	2.51	a	—
Cyanidin 3-O-galactoside	Anthocyanins	1.66	68.47	6.10
Cyanidin 3-O-glucoside (Kuromanin)	Anthocyanins	1.65	64.96	6.02
Delphinidin 3-O-glucoside (Mirtillin)	Anthocyanins	1.64	62.69	5.97
Pelargonidin 3-O-glucoside	Anthocyanins	1.59	49.81	5.64
(-)-Epiafzelechin	Flavanols	1.26	0.09	−3.53
Tricin-O-Hexoside-O-rhamnoside	Flavones	2.94	a	
Tilianin (Acacetin-7-O-β-D-glucoside)	Flavones	2.40	b	—
Diosmetin-6-C-glucoside	Flavones	2.16	a	—
Luteolin-6-C-2-glucuronylglucoside	Flavones	2.14	a	—
Diosmin	Flavones	2.12	a	—
Rhoifolin	Flavones	1.83	77.83	6.28
Acacetin-O-glucuronic acid	Flavones	1.72	b	—
Apigenin 7-rutinoside (Isorhoifolin)	Flavones	1.64	65.25	6.03
Luteolin-7-O-β-D-gentiobioside	Flavones	1.59	49.56	5.63
Acacetin-7-O-galactoside	Flavones	1.46	0.04	−4.75
Luteolin-O-rutinoside-O-rhamnoside	Flavones	1.27	0.08	−3.59
Quercetin coumarin glucoside	Flavones	1.17	9.04	3.18
Luteolin O-hexosyl-O-pentoside	Flavones	1.09	0.14	−2.79
Apigenin-6-C-2-glucuronylxyloside	Flavones oxyglycoside	1.12	7.36	2.88
Apigenin-6-C-β-D-xyloside-8-C-β-darabinoside	Flavones oxyglycoside	1.11	0.15	−2.78
Vitexin 2′′-O-β-L-rhamnoside	Flavones oxyglycoside	1.08	6.08	2.60
Kaempferol rha-glc-rha	Flavonols	2.86	b	—
Quercitrin	Flavonols	2.79	a	—
methylquercetin glu-rha	Flavonols	2.15	976.10	9.93
Kaempferol-3-O-glucoside-7-O-rhamnoside	Flavonols	1.07	0.17	−2.57
Kaempferol 3-O-rutinoside (Nicotiflorin)	Flavonols	1.02	0.20	−2.31
Afzelechin (3,5,7,4′-Tetrahydroxyflavan)	Isoflavones	1.33	0.06	−3.95
Tetragalloylglucose	Tannin	1.07	0.17	−2.53

a : Compounds only detected in ZJ;

b : Compounds only detected in YK-10.

Through S-plot analyses, 8 and 6 compounds were designated as marker metabolites in ZJ and YK-10, respectively (Fig. 2d). In the former, they were cyanidin 3-O-galactoside, cyanidin 3-O-glucoside (kuromanin), delphinidin 3-O-glucoside (mirtillin), petunidin 3-O-glucoside, pelargonidin 3-O-glucoside, apigenin 7-rutinoside (isorhoifolin), methylquercetin glu-rha, and diosmetin, and their levels were 49.81 times greater than in YK-10. In the latter, they were kaempferol 3-O-rutinoside (nicotiflorin), kaempferol rha-glc-rha, luteolin-O-rutinoside-O-rhamnoside, apigenin-6-C-D-xyloside-8-C-darabinoside, (–)-Epiafzelechin, and tetragalloylglucose, and their levels were 4.96 times greater than in ZJ.

### Flavonoid biosynthetic gene expression differences between ZJ and YK-10 shoots

Using Illumina HiSeq2000 sequencing and data analysis, 3,531 and 3,215 genes were identified as having higher and lower expression levels in ZJ compared with in YK-10, respectively. Additionally, 64, 22, 12, and 3 DEGs were assigned into the KEGG pathways of phenylpropanoid biosynthesis (ko00940), flavonoid biosynthesis (ko00941), anthocyanin biosynthesis (ko00942), and flavone & flavonol biosynthesis (ko00944), respectively, which are related to the biosynthesis of flavonoid.

The metabolic pathways of flavonoids biosynthesis were showed in Supplementary Fig. 1. Structural genes were annotated into these pathways, and among them, expression of 34 genes were different significantly between ZJ and YK-10. Compared with in YK-10, expression of 17 genes in ZJ were increased significantly, included 3 *PAL*s (CSA016076, 022024, 022025), 2 *DFR*s (CSA003949, XLOC_010242), 1 *ANS* (CSA011508), 1 *CHS* (CSA029775), 1 *SHT* (CSA023956), 1 *CCoAOMT* (XLOC_018193), 6 *UGT75L12/13* (CSA005544, 005545, 010001, 036671, 036672, 029026), and 2 *UGT94P1* (CSA007394, 008750). In addition, expression of other 17 genes decreased in ZJ significantly, included 1 *4CL* (CSA001434), 1 *DFR* (CSA035727), 1 *CHI* (CSA008261), 1 *FLS* (CSA008358), 1 *ANS* (CSA035767), 1 *ANR* (CSA011986), 1 *CHS* (CSA029773), 2 *LAR*s (CSA014943, XLOC_016774), 1 *F3′5′H* (CSA031792), 1 *SHT* (XLOC_018541), 2 *CCoAOMT* (CSA009706, 030460), 2 *UGT75L12*s (CSA008693, 028873), and 2 *UGT94P1*s (CSA005965, 026000) (Supplementary Fig. 1 and Table 2).

In a previous work, we obtained full-length transcriptomes of ZJ and YK-10 using PacBio Iso-sequencing (Chen, Shi *et al.* 2020). In total, 84, 46, 6, and 7 isoforms, respectively, in ZJ and 133, 37, 4, and 2 isoforms, respectively, in YK-10 were annotated to the ko00940, ko00941, ko00942, and ko00944 KEGG pathways (Supplementary Table 3). After an integrated analysis of transcriptomes, 34 isoforms (2 *PAL*s, 2 *C4H*s, 1 *TC4H*, 2 *4CL*s, 1 *CHI*, 3 *CHS*s, 1 *F3H*, 2 *F3′5′H*s, 3 *FLS*s, 1 *DFR*, 2 *LAR*s, 1 *ANS*, 1 *ANR*, 6 *UGT*s, 1 *C3′H*, 3 *CCoAOMT*s, and 2 *SHT*s) were finally chosen for further RT-qPCR analysis (Supplementary Table 4).

### Flavonoids biosynthetic enzyme expression differences between ZJ and YK-10 shoots

**Fig. 3.**
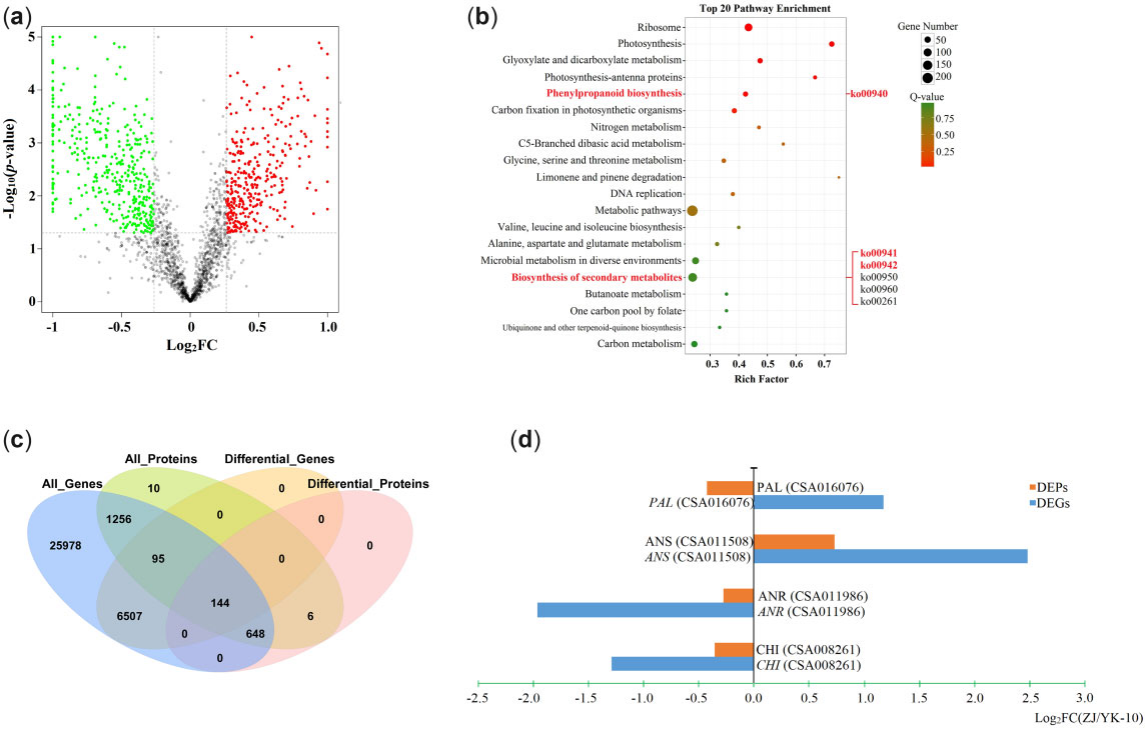
Results of the proteomics analyses. A volcano plot (a) and the top 20 KEGG enriched pathways (b) of DEPs; a venn diagram of all the proteins/genes and DEPs/DEGs (c); the expression profiles of DEP-DEG pairs involved in flavonoid biosynthesis (d).

Using a proteomics analysis, 4,186 proteins were identified. In comparison to YK-10, 415 and 383 proteins showed increased and decreased expression levels in ZJ (Fig. 3a). Among them, 25, 3, and 1 DEP were enriched int phenylpropanoid, flavonoid and anthocyanin biosynthesis pathways, respectively, and these pathways were among the 20 top enriched pathways (Fig. 3b). Among the 29 DEPs, 3 PALs, 1 CHI, 1 ANS, 1 ANR, and 1 UGT78A15 were directly involved in the biosynthesis of catechins and anthocyanins (Supplementary Table 5).

To further understand the functions of the DEPs in flavonoid biosynthesis, the correlations between DEPs and DEGs of ZJ and YK-10 were analyzed. The expression abundances of 144 DEPs were associated with the corresponding coding DEG levels, including 130 positively and 14 negatively correlated DEP-DEG pairs (Fig. 3c). Among them, 13 and 3 DEP-DEG pairs were enriched in the phenylpropanoid and flavonoid biosynthetic pathways, respectively. Only 4 DEP-DEG pairs were related to flavonoid biosynthesis. Among them, CHI (CSA008261), ANS (CSA011508), and ANR (CSA011986) showed the same expression trends as their corresponding DEGs, but PAL (CSA016076) showed a different expression trend (Fig. 3d; Supplementary Table 6a).

### Correlation analyses of DLMs and DEGs/DEPs

**Fig. 4.**
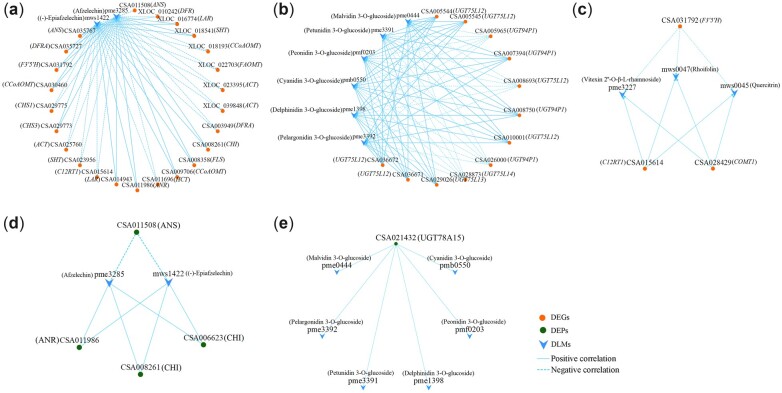
The correlation networks of DLMs and DEGs/DEPs in flavonoid (a, d), anthocyanin (b, e), flavone and flavonol (c) biosynthesis.

The correlation analyses of DLMs and DEGs revealed 3 shared KEGG pathways related to flavonoid biosynthesis. They were the flavonoid (2 DLMs and 24 DEGs), anthocyanin (6 DLMs and 12 DEGs), and flavone and flavonol (3 DLMs and 3 DEGs) biosynthetic pathways. Positive correlations were shown between the levels of some DEGs and DLMs, including 12 DEGs (1 *SHT*, 2 *CCoAOMT*s, 1 *CHI*, 1 *CHS*, 1 *F3′5′H*, 1 *FLS*, 1 *DFR*, 2 *LAR*s, 1 *ANS*, 1 *ANR*) and 2 flavanols [(−)-epiafzelechin, afzelechin], 8 DEGs (5 *UGT75L12*, 1 *UGT75L13*, 2 *UGT94P1*), and 6 anthocyanin 3-O-glucosides (pelargonidin/delphinidin/cyanidin/peonidin/petunidin/malvidin 3-O-glucosides), and 2 DEGs (1 *C12RT1*, 1 *COMT1*) and 3 flavone glycosides (quercitrin, rhoifolin, vitexin 2′′-O-β-L-rhamnoside) (Fig. 4, a–c; Supplementary Table 6b).

Flavonoid biosynthetic (2 DLMs and 4 DEPs) and anthocyanin biosynthetic (6 DLMs and 1 DEP) pathways were both annotated in the DLM and DEP correlation analysis. The expression of UGT (CSA021432) was positively correlated with the levels of 6 anthocyanin 3-O-glucosides. The DEPs expression of CHI (CSA008261, 006623) and ANR (CSA011986) were positively correlated with the flavanol accumulations of (−)-epiafzelechin and afzelechin, whereas the expression of ANS (CSA011508) was negatively correlated with their levels (Fig. 4, d and e; Supplementary Table 6c). Interestingly, the expression of *CHI* (CSA008261) and *ANR* (CSA011986) showed positive correlation, while expression of *ANS* (CSA011508) showed negative correlation with the abundances of (−)-epiafzelechin and afzelechin, respectively.

### Integrated analysis of transcriptomic, proteomic and metabolomic data

**Fig. 5.**
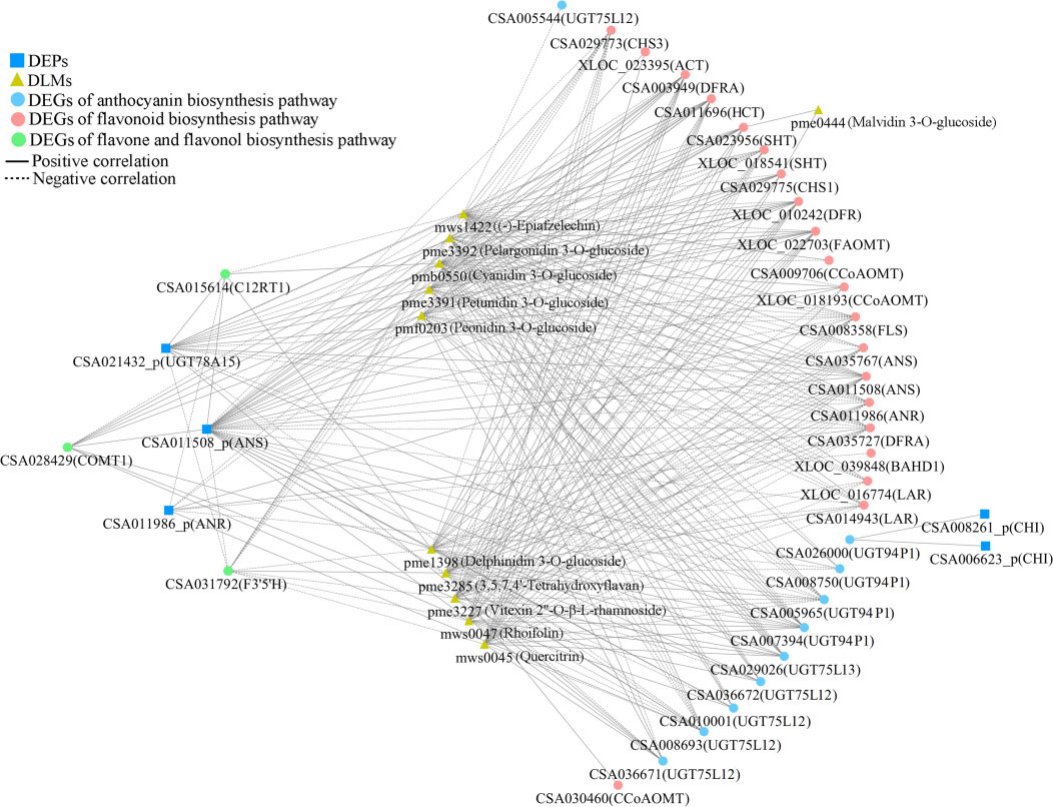
The correlation network of DEGs, DEPs and DLMs related to flavonoids biosynthesis.

To gain a deeper understanding of the flavonoid metabolic mechanisms, the transcriptome-proteome-metabolome correlations were analyzed on the bases of DEGs-DEPs-DLMs involved in the flavonoid pathway (Fig. 5; Supplementary Table 7). In the flavonoid biosynthetic pathway, the expression of most DEGs showed opposite correlations with the expression of ANS (CSA011508) and accumulations of (−)-epiafzelechin and afzelechin. The accumulations of (−)-epiafzelechin and afzelechin presented negative correlations with the expression of ANS (CSA011508) but had no direct correlation with that of ANR (CSA011986). The DEGs expression of *DFR* (CSA035727) and *FLS* (CSA008358) showed negative associations with the DEP level of ANS, while they showed positive association with DEP level of ANR and DLMs accumulations of (−)-epiafzelechin and afzelechin. However, the expression of *DFR* (CSA003949) and *HCT* (CSA011696) positively correlated with level of ANS but negatively correlated with level of ANR, (−)-epiafzelechin, and afzelechin.

In the anthocyanin biosynthetic pathway, the expression of 8 *UGT* genes (*UGT94P1* and *UGT75L12/13*) correlated with the accumulation of 5 anthocyanin 3-O-glucosides. Only the expression of 4 DEGs (2 *UGT94P1* and 2 *UGT75L12*) directly correlated with the levels of DEP UGT78A15 (CSA021432) and anthocyanin 3-O-glucosides at the same time. The levels of *UGT94P1* (CSA005965) and *UGT75L12* (CSA008693) were negatively correlated with the levels of UGT78A15 and anthocyanin 3-O-glucosides, but those of the *UGT94P1* (CSA007394) and *UGT75L12* (CSA036671) showed opposite correlations. In addition, the expression of *UGT94P1* (CSA026000) was positively correlated with the level of the DEP CHI (CSA008261, 006623), but it showed no correlation with the abundances of anthocyanin 3-O-glucosides. In the flavone and flavonol biosynthetic pathway, the expression of the DEGs *F3′5′H* (CSA031792) and *CCoAOMT1* (CSA028429) presented negative and positive correlations, respectively, with the flavone glycosides levels of rhoifolin, quercitrin, and vitexin 2′′-O-β-L-rhamnoside. There was no DEP level in this pathway was associated with DEG and DLM levels. However, the DEG expression of *C12RT1* (CSA015614) showed correlations with the DEP and DLM levels in other pathways. Generally, the connections among the DEG, DEP, and DLM levels were not limited to the same pathway.

### Verification of the correlations between gene expression and flavonoid contents

**Fig. 6.**
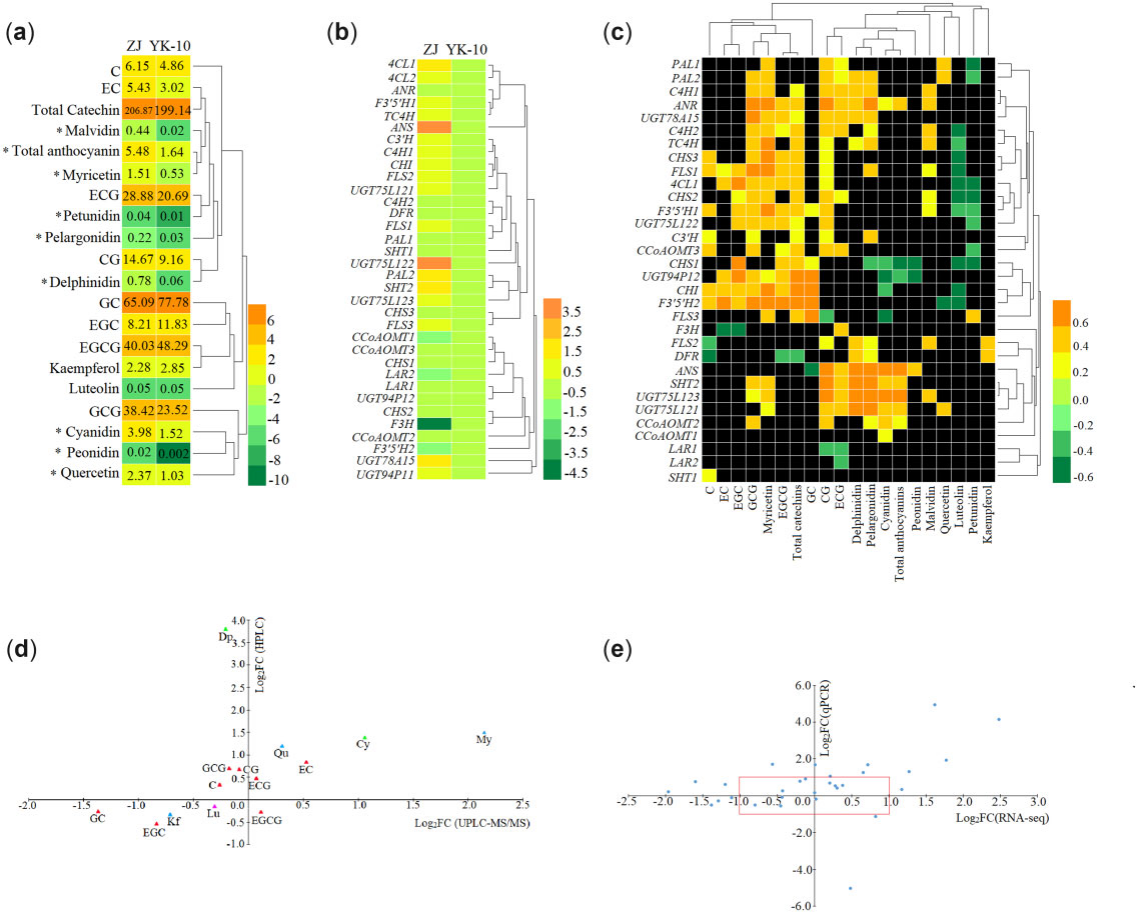
The HPLC analyses of the contents of 18 flavonoids (a, c), RT-qPCR analyses of 34 related biosynthetic genes (b, d) and correlation analyses between them (e). *Note*: a: the numbers represent contents of compounds (mg/g); * represents significant differences (log_2_FC > 1 or log_2_FC < −1).

To verify the UPLC-MS/MS and RNA-seq results, 18 flavonoids and 34 biosynthetic genes in ZJ and YK-10 shoots were detected using HPLC and RT-qPCR, respectively (Fig. 6, a and b). Among them, 14 flavonoids and 30 genes had been determined using omics technology. In ZJ/YK-10, the contents of 9 flavonoids showed similar trends in the metabolomics analysis and HPLC measurements. Additionally, the expression patterns of 20 genes showed similar trends in the RNA-seq analysis and RT-qPCR. Additionally, the differences in flavonoid and genes levels were mostly not significant, as indicated by the absolute log_2_FC values being less than one in the omics data (Fig. 6, c and d). The HPLC results showed that the contents of Cy and 6 anthocyanins in ZJ were significantly greater than in YK-10, but the contents of 8 catechins showed no significant differences. Interestingly, the expression levels of most genes were higher in ZJ than in YK-10.

Correlation analyses showed that the most significant correlations were positive, and the expression of most early genes played positive roles in catechin accumulation. The expression of *ANS*, which had a 17 times greater level in ZJ compared with in YK-10, positively correlated with the Cy and total anthocyanin accumulations. The expression of *ANR*, *UGT75L121/123*, *SHT*, and *CCoAOMT2* also promoted anthocyanin biosynthesis. Additionally, the expression of *DFR* and the contents of EGCG showed negative correlations. The expression of *ANS* and the content of GC, as well as the expression of *CHS1*, *CHI*, and *FLS3* and the level of Cy, showed negative correlations (Fig. 6e).

## Discussion

Using the 2^nd^ transcriptome, 101 DEGs of ZJ/YK-10 encoding flavonoid biosynthetic enzymes were identified. The expression levels of 2 *DFR*s, 1 *ANS* (CSA011508), and 8 *UGT*s in ZJ were significantly higher than in YK-10. However, the expression of *ANR* (CSA011986) presented the opposite profile. Furthermore, the expression of ANS (CSA011508) and ANR (CSA011986) showed consistent trends with their encoding genes, and negatively and positively correlated with the DLMs of flavanols. Therefore, we speculate that the DEGs with high FPKM values, especially *ANS* (CSA011508) and *ANR* (CSA011986), play key roles in the accumulation and distribution of catechins and anthocyanins in ZJ. The high expression of *ANS* (CSA011508) and low expression of *ANR* (CSA011986) stimulate the accumulation of anthocyanins by reducing the conversion to catechins (Seitz *et al.* 2003; Mei *et al.* 2020).

In the DLM-DEG and DLM-DEP integration analyses, there were more correlated DLM-DEG pairs than DLM-DEP pairs involved in the flavonoids pathways. There were no interacting DLM-DEP pairs in the flavone and flavonol biosynthetic pathway, which may indicate that flavonoid accumulation is regulated mainly at the gene level but not at the protein level. The contents of 6 anthocyanidin 3-O-glucosides, 3 flavone glycosides and 2 flavanols were correlated with DEG/DEP expression. The 2 flavanols, (−)-epiafzelechin and afzelechin, are dimer and trimer units of propelargonidin, which is a proanthocyanidin (Omar *et al.* 2011). The expression of *LAR* (CSA014943, XLOC_016774) showed positive correlations with the contents of (−)-epiafzelechin and afzelechin. In our research, the lower level of *LAR* in ZJ was consistent with the reported results (Hossain *et al.* 2018; Mei *et al.* 2020), but the displayed correlations with (−)-epiafzelechin and afzelechin were noted for the first time. Therefore, we speculated that the lower expression of *LAR* resulted in the lower accumulations of proanthocyanidins in ZJ than in YK-10.

In addition, the importance of the gene *UGT* in anthocyanin biosynthesis was demonstrated once again by a multi-omics correlation analysis (Fang *et al.* 2021). *UGT75L12* and *UGT78A15* are responsible for flavonol 7-O-glucosides and flavonol 3-O-galactosides biosynthesis (Cui *et al.* 2016). *UGT94P1* converts volatile organic compounds into β-primeverosides by sequential xylosylation (Ohgami *et al.* 2015). In our research, the expression of *UGT94P1* (CSA005965, 007394) and *UGT75L12* (CSA008693, 036671) showed direct correlations with the expression of UGT78A15 (CSA021432) and accumulation of anthocyanin 3-O-glucosides. Furthermore, the expression levels of *UGT75L12* (CSA036671), *UGT94P1* (CSA007394), and the UGT78A15 (CSA021432) enzyme were positively correlated with the contents of anthocyanin 3-O-glucosides. Thus, the higher expression levels of genes *UGT75L12* (CSA036671), *UGT94P1* (CSA007394), and enzyme UGT78A15 (CSA021432) promoted a greater accumulation of anthocyanin 3-O-glucosides in ZJ/YK-10.

**Fig. 7.**
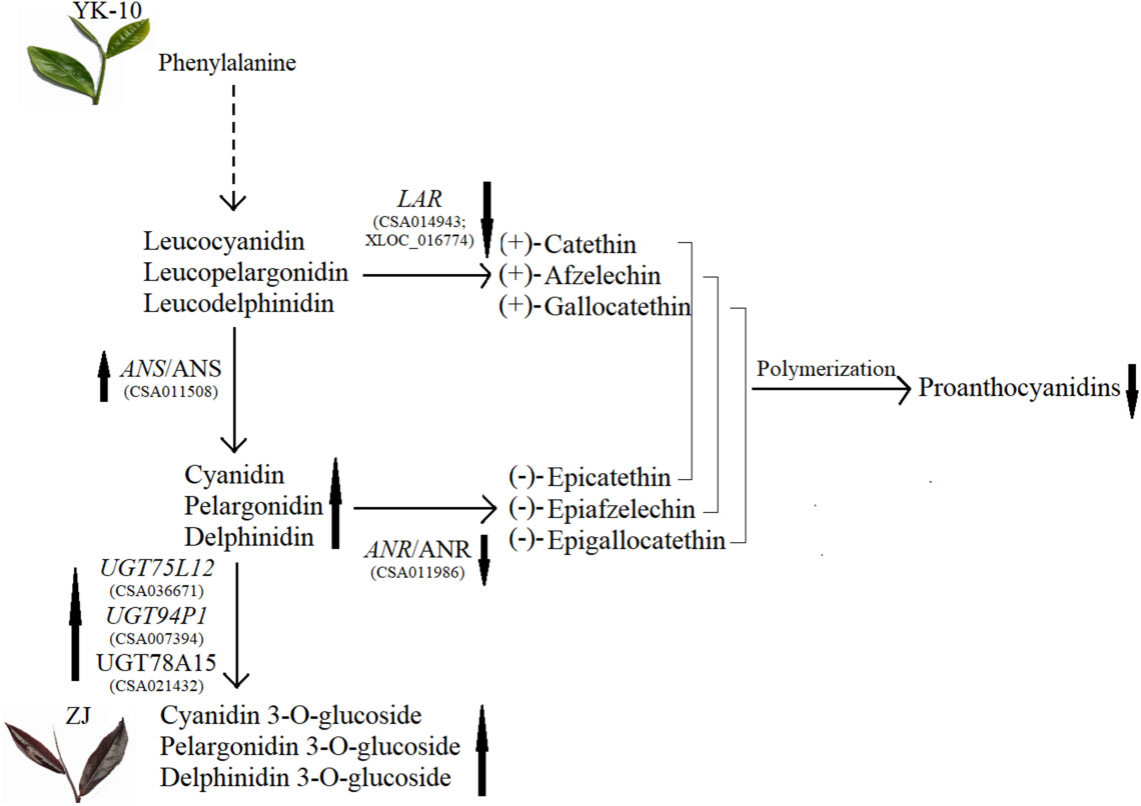
The proposed model of molecular mechanisms of purple-color formation in tea shoots. *Note*: the orange and green ellipse represents DEG and DEP; the red arrows represent the DEG and DEP trends; the green arrows represent the DLM trends.

These special DEGs [*ANS* (CSA011508), *ANR* (CSA011986), *LAR* (XLOC_016774), *UGT75L12* (CSA036671), *UGT94P1* (CSA007394)] and DEPs [ANS (CSA011508), ANR (CSA011986), UGT78A15 (CSA021432)] may play indispensable roles in the molecular mechanisms associated with the purple color in ZJ shoots. The competition for reaction substrates may responsible for the opposite expression profiles between *ANS* and *LAR*, *ANR* genes, and ANS and ANR enzymes (Mei *et al.* 2020). Under the collective effects of these DEGs and DEPs, the ZJ shoots present as purple with the decrease in proanthocyanidins and increase in anthocyanins. The anthocyanins were further converted into anthocyanins glucosides, which are more stable structures (Liu *et al.* 2018) (Fig. 7).

In ZJ shoots, the total catechins, and especially anthocyanin, concentrations were higher than in YK-10 shoots, which corresponds to the quality characteristics of ZJ as described previously (Yang *et al.* 2009). Among the catechins, the content of EGCG analyzed by HPLC in ZJ was less than in YK-10, which was opposite of the UPLC-MS/MS data. This may be because the heating during the extraction process promotes the isomerization and reduction of EGCG (Jiang *et al.* 2021). The higher concentration of GCG in our experiment, which is a transisomer of EGCG, was also probably related to the extraction method.

The RT-qPCR results confirmed that the higher expression levels of most biosynthetic genes were important reasons for higher catechin and anthocyanin levels in ZJ/YK-10(Aza-González *et al.* 2013). The roles of early genes in anthocyanin accumulation have been shown in other purple plants (Wang, Luo *et al.* 2019), and the correlations between Dp and Pg abundance and early gene expression levels were almost positive in our study. The high gene expression level and catalytic activity of UGT promotes flavonoid accumulation (He *et al.* 2018), and 5 *UGT* isoforms with higher values in ZJ were verified by HPLC. The differential expression levels of *ANS* (CSA011508), *LAR* (CSA014943), and *UGT* (CSA036672) identified by RT-qPCR were consistent with the transcriptome predictions. Furthermore, the *ANS* level correlated negatively with GC content but positively with Cy, Dp, Pg and total 6 anthocyanins content in our study. This was consistent with *ANS* expression promoting the anthocyanin accumulation in *Solanaceous* vegetables (Liu *et al.* 2018). This indicates that the proposed model of purple-color formation in tea shoots is credible to some extent.

In general, we attempted to understand more clearly the molecular mechanisms behind the flavonoid differences that exist in tea shoots showing color variations using omics integrated analyses. The key *ANS*, *ANR*, *LAR*, *UGT75L12*, and *UGT94P1* genes and the key ANS, ANR, UGT78A15 enzymes were predicted to play essential roles in anthocyanin accumulation. This demonstrated that the anthocyanin accumulation was positively associated with *ANS* and *UGT75L12* expression as determined by HPLC and RT-qPCR combined analyses. However, the results of the RT-qPCR and HPLC experiments were not totally consistent with those of the omics analyses. Furthermore, the expression profiles of flavonoid biosynthetic genes are not only influenced by many environment factors, such as light (Li *et al.* 2020) and abiotic stresses (Gai *et al.* 2020), they are also regulated at the transcriptional and translational levels (Wu *et al.* 2020). The regulator genes of transcription factors like MYB, bHLH, WD40 and complex of them involving in the flavonoid biosynthesis, and their regulation mechanism through target genes are complex but worth to study. It is the most important work in progress for our team to explore and verify the regulator genes using transgenic and other biotechnologies.

## Supplementary Material

jkac297_Supplementary_Figure_S1

jkac297_Supplementary_Table_S1

jkac297_Supplementary_Table_S2

jkac297_Supplementary_Table_S3

jkac297_Supplementary_Table_S4

jkac297_Supplementary_Table_S5

jkac297_Supplementary_Table_S6

jkac297_Supplementary_Table_S7

jkac297_Supplementary_Table_S8

## Data Availability

All data generated or analyzed during this study are included in this published article and its Supplementary Materials. The raw data of transcriptome and proteome have been respectively deposited to Sequence Read Archive database (https://submit.ncbi.nlm.nih.gov/about/sra/) (PRJNA524304) and iProX database (https://www.iprox.org/) (PXD036754). The qualitative and quantitative analysis result of all flavonoids identified by target metabolome was uploaded as Supplementary Table 8. Supplemental material is available at G3 online.
