# Supplementary material for: Multiomics analysis of the mechanisms behind flavonoid differences between purple and green tender shoots of Camellia sinensis var. assamica
Source: G3 (Bethesda). 2022 Nov 7;13(2):jkac297. doi: 10.1093/g3journal/jkac297 (PMC9911070; doi:10.1093/g3journal/jkac297)
Supplement: jkac297_Supplementary_Figure_S1 [file jkac297_supplementary_figure_s1.docx]

Figure S1


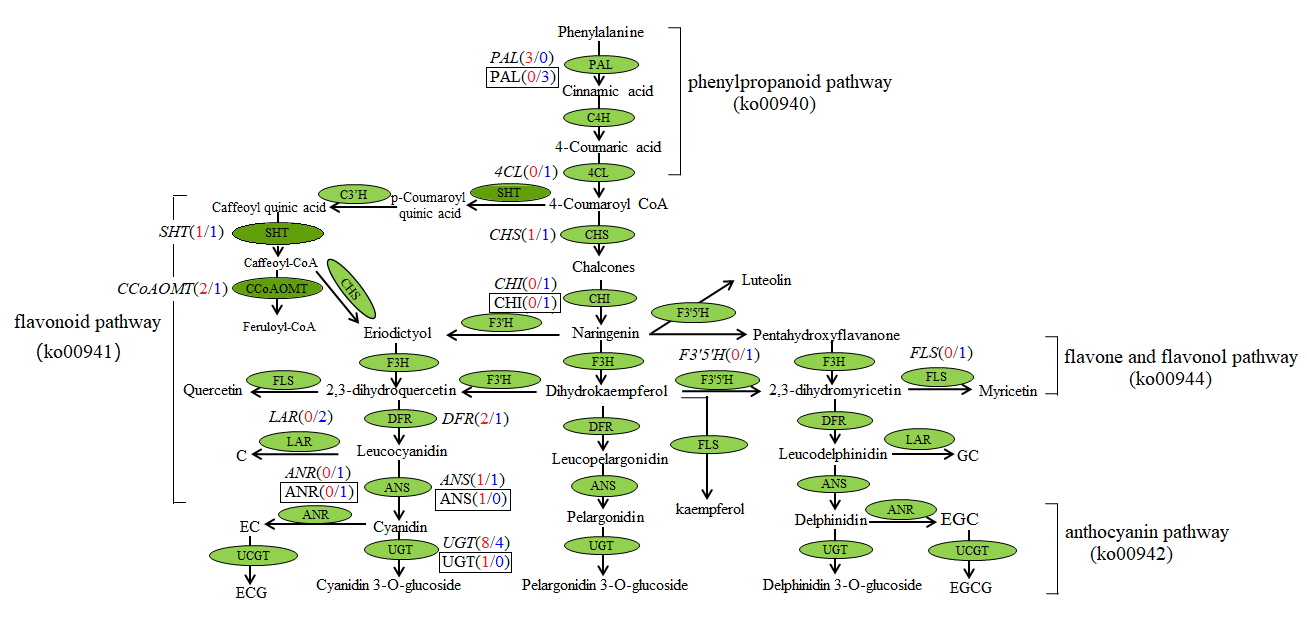


**Figure S1 The flavonoids biosynthesis pathways**

Note：Red and blue number represent up-regulated and down-regulated; Non-italic and Italic represent DEPs and DEGs, respectively
